# The Sweet Side of Fungal Infections: Structural Glycan Diversity and Its Importance for Pathogenic Adaptation

**DOI:** 10.3390/medicines9060037

**Published:** 2022-06-16

**Authors:** Israel Diniz-Lima, Leonardo Marques da Fonseca, Jhenifer Santos dos Reis, Marcos André Rodrigues da Costa Santos, Kelli Monteiro da Costa, Carlos Antonio do Nascimento Santos, Pedro Marçal Barcelos, Kamila Guimarães-Pinto, Alessandra Almeida Filardy, Marco Edilson Freire-de-Lima, Debora Decote-Ricardo, Alexandre Morrot, Celio Geraldo Freire-de-Lima, Leonardo Freire-de-Lima

**Affiliations:** 1 Instituto de Biofisica Carlos Chagas Filho, Universidade Federal do Rio de Janeiro, Rio de Janeiro 21941-170, Brazil; israel@biof.ufrj.br (I.D.-L.); lfonseca@biof.ufrj.br (L.M.d.F.); jheniferreis@biof.ufrj.br (J.S.d.R.); rodrigues8mr@biof.ufrj.br (M.A.R.d.C.S.); kellimc85@gmail.com (K.M.d.C.); cansantos.bio@gmail.com (C.A.d.N.S.); pedrombarcelos73@gmail.com (P.M.B.); leolima@biof.ufrj.br (L.F.-d.-L.); 2 Instituto de Microbiologia Paulo de Goes, Universidade Federal do Rio de Janeiro, Rio de Janeiro 21941-170, Brazil; guimaraespkamila@micro.ufrj.br (K.G.-P.); filardy@micro.ufrj.br (A.A.F.); 3 Instituto de Química, Departamento de Química Orgânica, Universidade Federal Rural do Rio de Janeiro, Rio de Janeiro 23890-000, Brazil; marco@ufrrj.br; 4 Departamento de Microbiologia e Imunologia Veterinária, Instituto de Veterinária, Universidade Federal Rural do Rio de Janeiro, Rio de Janeiro 23890-000, Brazil; decotericardo96@gmail.com; 5 Instituto Oswaldo Cruz, FIOCRUZ, Rio de Janeiro 21040-360, Brazil; alexandre.morrot@ioc.fiocruz.br

**Keywords:** fungal infection, glycoconjugates, immune system, cytokines

## Abstract

Fungal infections are the most common secondary infections in debilitated individuals in a state of chronic disease or immunosuppression. Despite this, most fungal infections are neglected, mainly due to the lower frequency of their more severe clinical forms in immunocompetent individuals with a healthy background. However, over the past few years, several cases of severe fungal infections in healthy individuals have provoked a change in the epidemiological dynamics of fungal infections around the world, both due to recurrent outbreaks in previously infrequent regions and the greater emergence of more pathogenic fungal variants affecting healthy individuals, such as in the *Cryptococcus* genus. Therefore, before the arrival of a scenario of prevalent severe fungal infections, it is necessary to assess more carefully what are the real reasons for the increased incidence of fungal infection globally. What are the factors that are currently contributing to this new possible epidemiological dynamic? Could these be of a structural nature? Herein, we propose a discussion based on the importance of the virulence factors of glycoconjugate composition in the adaptation of pathogenic fungal species into the current scenario of increasing severity of these infections.

Currently, fungal infections are a major cause of comorbidity globally [1]. Indeed, pathogenic fungi contribute to a large portion of deaths in immunocompromised individuals or as a secondary disease [2]. Historically, the pathogenic species of the genus *Cryptococcus*, *Crytococcus neoformans* and *Cryptococcus gattii* were fungi known to chronically establish a silent infection with difficult diagnosis in the early stages of disease progression [3]. This is mainly complicated by the low spectrum of effective antifungal drugs for the most serious infections [4], especially when associated with the spread of the pathogenic microorganism to critical host tissues such as the central nervous system where it can lead to severe fungal meningoencephalitis [5,6]. In particular, there are indications that the dynamics of infections caused by *Cryptococcus* spp. have been modified over the years. Previously, this pathogenic fungus was mostly associated with warmer climate areas where its environmental incidence was higher [7], being found in forests and plant material in tropical or subtropical morphoclimatic domains [8]. However, there has been a surge of cases over the years in previously uncommon places [9,10,11]. In addition, the highest global incidence of cryptococcosis has always been associated with regions with a history of human immunodeficiency virus (HIV) infections, such as sub-Saharan Africa [12]. The prevalence of a virus that causes severe immunodepression, as is the case with acquired immunodeficiency syndrome (AIDS), creates space for opportunistic infections to thrive in weakened hosts [13]. Consistently, the AIDS crisis at the end of the 20th century was crucial for the increased number of secondary infections caused by *C. neoformans* [14]. This phenomenon demonstrated the great epidemiological proportions that a fungal infection can reach, and exposed the world’s lack of preparation to deal with any severe opportunistic fungal infection outbreaks. In addition to the species of the genus *Cryptococcus*, debilitated individuals are also affected by several other genera of fungi [15], such as the genus *Aspergillus*, which is responsible for cases of severe pneumonia in immunologically weakened hosts, promoting a high mortality rate and high number of hospitalizations worldwide [16]. Furthermore, some pathogenic species of fungus can affect healthy individuals, causing serious infections, for example, the species of *C. gattii* [17]. Interestingly, this species has stood out for its epidemiological dynamics, with more cases reported in urban environments, increasingly distancing itself from its primary incidence in wild habitats [18,19,20,21]. In addition, there is the example of the genus *Candida* spp. which, despite being a commensal fungi on body surfaces, can cause serious superficial infections in the skin and mucous membranes of both immunocompromised and immunocompetent individuals [22] and may even cause severe forms of systemic dissemination [23]. Moreover, it is also worth factoring in the COVID-19 pandemic and its more recent outbreaks. The high mortality among COVID-19 patients also suffering from fungal co-infections emphasizes the necessity to take those pathologies with great concern [24]. Multiple countries related an increase in the cases of fungal co-infections in severe COVID-19 patients [25]. The majority of the cases were associated with pulmonary aspergillosis [26], pneumocystis pneumonia [27], cryptococcal pneumonia [28], *C. laurentii* endophthalmitis [29] and *C. neoformans* meningoencephalitis in an immunocompetent patient [30], and also some were related to the outbreaks of the rare mucormycosis infection in India, which is often life-threatening [31]. The susceptibility in these patients can be justified mainly by the use of corticosteroids, which increases the risk of a secondary infection by suppressing an already debited immune system, opening a space for fungal infection in patients with a weakened antifungal immunity [32]. Indeed, in some cases, critically-ill COVID-19 patients appear to be at low risk for fungal infections when compared to those who have undergone corticosteroid treatment [33].

However, a question arises in the midst of all these infections caused by fungi: what are the real contributing factors leading to a greater spread of pathogenic fungal species? Are their differences of an environmental nature or due to evolutionary traits? Indeed, the intrinsic susceptibility of permissive individuals to fungal infection alone does not justify the increase in fungal dissemination, since immunocompetent individuals can be affected by many of these infections [34]. In fact, there are several factors that systematically contribute to minimize the geographic restrictions of fungi and increase the prevalence of these diseases. It is believed that global warming contributes to the change in dynamics of animal populations, including fungus-carrying species, as is the case for *Cryptococcus* spp., which is strongly associated with birds. [35]. The deforestation of wild environments with the ever-expanding limits of modern agricultural activities and the extensive reforestation of invasive tree species such as the *Eucalyptus* also play a pivotal role in favoring the dispersion of environmental fungus species to regions outside their original niche [36]. This geographical displacement pressure can result in evolutionary adaptation of pathogenic fungal species in order to establish themselves environmentally. Therefore, it is also suggested that the evolutionary distance of fungal species is also due to the dissemination into different geographic regions [37]. This dynamic is critical for the differences in the molecular composition of these different intra-species or intra-genus variations of fungi [38]. The evolutionary pressure generated by the need for adaptation of fungal species may contribute to the emergence of increasingly resistant strains. For instance, a hypervirulent strain of *C. deuterogattiii* (R265), genetically originated from Brazil [39], gained distinction by causing the infamous epidemiological outbreak of severe cryptococcosis in human hosts and in animal wildlife on the Pacific North Coast in Canada [40], which continues to spread along the US west coast and into California [41]. In addition, a high radiation tolerance for some strains of *Cryptococcus* in the Chernobyl Exclusion Zone—UA—has been described. Such specimens exhibit highly melanized yeast morphology associated with resistance mechanisms against environmental stress [42]. Hence, there is likely a strong relationship between the adaptation of the fungus species and the modifications of the molecular constituents, mainly in the fungal virulence factors.

A substantial portion of the main virulence factors of fungi are composed of glycoconjugates [43,44,45,46]. These polysaccharides, mostly found in the cell wall, play an important structural role, and many are conserved among different species of fungi [47]. One of the main components present in the fungal cell wall is the *β*-glycans, with the *β*-(1,3)-glycan being the most abundant in *C. albicans* species and *β*-(1,6)-glycan in *C. neoformans* species [48,49]. These components provide structural stability to the fungal cell and a way of interacting with host cells. For instance, disrupting the binding of *β*-(1,3)-glycan to dectin-1 and CD11b inhibits macrophage phagocytosis [50]. Interestingly, it has already been observed that glycans of higher molecular weight elicit greater activation of macrophages, thus demonstrating that the potential for cell activation is related to glycoconjugate size [51]. The same behavior has already been reported on another important component for the structuring of the fungal cell, the chitin molecule, composed of long chains of *N*-acetylglucosamines [52]. Although large chitin fragments are inert, intermediate and small ones are capable of inducing cytokine production in vivo and in vitro. Furthermore, different sized fragments are capable of interacting with different host receptors activating distinct signaling pathways causing intermediate fragments to upregulate TNF-α release, while smaller fragments stimulates both TNF-α and IL-10, indicating that mismatched sized chitin fragments lead to different immune system behavior [53]. Interestingly, the percentage of chitin in the cell wall of fungi varies greatly according to their morphology, ranging from 1% to 2% in yeasts, and reaching up to 15% in filamentous fungi [54]. Indeed, the chitin levels vary through fungi environmental adaptation, as is the case of the Aspergillus species. This pathogenic fungus enhances its chitin production in either hypoxic environments or higher glucose conditions, and this is directly related to its ability of regulating metabolism through the hexosamine biosynthetic pathway [55,56,57]. Thus, the increase in the chitin composition is due a metabolic shift that culminates in fungal resistance to external stress both in the environment and during pathogenesis [58]. In addition to these components associated with the cell wall, there are mannan glycoconjugates found in *C. albicans* and galactomannans present in *A. fumigatus* [59]. Mannans have a more fluid structure in relation to *β*-glucans and chitin, covering the outermost part of the cell wall, thus, despite not greatly influencing the structural stiffness of the fungal cell, they block the interaction of the inner *β*-glucans with immune system receptors [60]. Actually, different strains of *C. albicans* that are deficient on the *N*- and *O*- mannan glycosylation process present low binding affinity to innate immune cells, such as neutrophils, resulting in a low phagocytic capacity and ensuing lack of pathogen restraint [61]. Indeed, during infection, *C. albicans* also has the capability to regulate its mannan remodeling through environmental induction, thus controlling its *β*-glucan exposure and modulating immune recognition [62,63]. Galactomannans are polymers composed of mannans and galactofuranoses that are also found anchored to glycosylphosphatidylinositol (GPI) molecules in the cell membrane of *A. fumigatus* [64]. This component is involved in the activation of phagocytes via DC-SIGN and Dectin-2 receptors, which may lead to the production of pro-inflammatory cytokines such as TNF-α [65,66]. In addition to these glycoconjugates, there are the glucuronoxylomannan (GXM) and glucuronoxylomannogalactan (GXMGal) molecules found uniquely in the polysaccharide capsule of *Cryptococcus* spp. [67]. This genus of pathogenic fungus has the unique feature of producing these virulence factors that are considered the most important ones for the pathogenic *Cryptococcus* species [68], mainly because of their pronounced immunomodulatory potential in the immune system cells [69]. It has been shown that GXM with larger dimensions elicit greater production of nitric oxide by macrophages and that fractions of different molecular weights of GXM are functionally distinct for the induction of cytokine production in vitro and in vivo [70,71]. Both GXM and GXMGal induce phagocyte apoptosis and the expression of Fas and Fas-L receptors that are associated with the activation of cell death induction pathways [72]. Interestingly, GXM elicits immunoregulatory responses, inducing IL-10 production in immune cells [73], downregulation of molecules associated with antigenic presentation in phagocytes, such as MHCII and CD86 [74], inhibition of TNF-*α*, IL-1*β* and IFN-*γ* production [75,76] and inhibition of the release of neutrophil extracellular traps (NET) [77]. Conversely, GXMGal elicits a pro-inflammatory profile and activation of antifungal responses with the induction of NET release [77], increased expression of MHCII and CD86 in phagocytes and the induction of IL-6 and IL-17 production, important cytokines for triggering antifungal immunity [78]. Furthermore, a possible association can be made with the increased expression of galectin-3 found in individuals with *Cryptococcus* spp. infection with the presence of galactose in the backbone structure of the capsular components of the fungus, so it ends up contributing to the fight against infection by cellular activation via the interaction of the capsular components with galectin-3 [79]. The range of interactions of fungal glycoconjugates with the immune system cells is quite diverse. Therefore, some of these effects on cytokine production, receptor expression and modulation of some components of the immune system are summarized in Table 1.

All these possible interactions with the immune system, especially when dealing with the direct interaction with glycoconjugates, suggest the possibility of adaptive mechanisms to pathogenic fungi that may emerge and favor their virulence. For example, the existence of hypervirulent strains of *Cryptococcus* spp., as in the example of the hypervirulent strain of *C. gattii* JP02, which has fewer *O*-acetylations in its GXM than the hypervirulent strain H99 of *C. neoformans*, has the characteristic which causes JP02 to disfavor a pro-inflammatory cytokine profile [90]. In addition to *O*-acetylations, xylosylations that are considered molecular patterns associated with binding affinity with the immune system, complement proteins and antibodies, presenting distinct abundance profiles amongst different strains of *Cryptococcus* [91,92]. Our research group has recently described a high rate of *O*-acetylations in *β*-galactofuranose residues of the GXMGal structure of *C. neoformans* grown in capsular growth-inducing medium, demonstrating the high presence of important binding sites in this polysaccharide structure that confer potential immunobiological activity [93,94]. Furthermore, the glycoinositolphosphorylceramides (GIPC) biosynthetic pathway, a class of glycolipids, produces essential molecules for the viability and virulence of *Cryptococcus* spp., specifically the presence of groups with xylose branches in the structure of GIPCs [95]. Thus, as it has already been described that mutant strains of *C. neoformans* deficient in the final production of Xylose, NE178 and NE321 are unable to complete the assembly of GIPCs [96]. Therefore, these modifications only reinforce the importance of the fine regulation of the fungal glycoconjugates synthesis and the interference that these components have on the virulence of these pathogenic fungi. Furthermore, a better understanding of the changes in the adaptation of the virulence factors allows the development of new pharmaceutical strategies to combat fungal diseases, since there is currently a low drug spectrum for the treatment of fungal infections [97]. Finally, the answer to the initial question is based precisely on the fact that fungi rely on the adaptation of their biochemical machinery, as the synthesis of their virulence factors depends on different environmental conditions, both as a free-living organism or as a pathogen. Thus, changes in the morphology and structural composition of fungal species tend to contribute to the diversity and pathogenicity of the various existing strains of pathogenic fungi.

## Figures and Tables

**Table 1:** Immunomodulatory role of some pathogenic fungi glycoconjugates.

Pathogen	Glycoconjugate	Immunomodulatory Role	References
*Cryptococcus* spp.	glucuronoxylomannan	↑NO; ↑apoptosis; ↑Fas; ↑FasL; ↑IL-10; ↓MHCII; ↓CD86; ↓TNF-*α*; ↓IL-1*β*; ↓IFN-*γ*; ↓NET release	[72,73,74,75,76,77]
	glucuronoxylomannogalactan	↑apoptosis; ↑Fas; ↑FasL; ↑NET release; ↑MHCII; ↑CD86; ↑IL-6; ↑IL-17	[72,77,78]
*Pneumocystis* spp.	*β*-glucan	↑IL-23; ↑IL-6; ↑IL-17; ↑IL-22; ↑IL-8; ↑MIP-2; ↑IL-1*β*; ↑TNF-*α*; ↑Fas; ↑FasL	[80,81,82]
*C. albicans*	mannan	↑IL-12; ↑IL-6; ↑TNF-*α*; ↑IL-17; ↑IL-2; ↑IL-4; ↑IFN-*γ*	[83,84,85,86]
*Aspergillus* spp.	galactomannan	↑TNF-α; ↑IL-6; ↑IL-1Ra	[66,87]
	*β*-glucan	↑IL-1*α*; ↑IL-1*β*; ↑IL-22; IL-4, IL-13, ↑IFN-*γ*; ↑IL-17A	[88,89]

Some glycan molecules from pathogenic fungi and their effects on cytokine production, receptor expression and modulation of immune components are summarized with referenced articles. ↑ upregulation. ↓ downregulation.

## Data Availability

Not applicable.
